# Estimated Dietary Exposure to Mycotoxins after Taking into Account the Cooking of Staple Foods in Japan

**DOI:** 10.3390/toxins5051032

**Published:** 2013-05-21

**Authors:** Hisako Sakuma, Yasushi Watanabe, Hiroko Furusawa, Tomoya Yoshinari, Hajime Akashi, Hiroshi Kawakami, Shiro Saito, Yoshiko Sugita-Konishi

**Affiliations:** 1 National Institute of Health Sciences, 1-18-1, Kamiyoga, Setagaya-ku, Tokyo 158-8501, Japan; E-Mails: sakuma.hisako.xhrqx@showadenko.com (H.S.); wata36-7@mse.biglobe.ne.jp (Y.W.); h-furusawa@nihs.go.jp (H.F.); t-yoshinari@nihs.go.jp (T.Y.); 2 QE Center Research and Development, Quality Assurance Division, Nisshin Seifun Group INC., Tsurugaoka, Fujimino, Saitama 356-8511, Japan; E-Mail: akashih@mail.ni-net.co.jp; 3 Faculty of Home Economics, Kyoritsu Women’s University, Hitotsubashi, Chiyoda, Tokyo 101-0003, Japan; E-Mail: hirokami@kyoritsu-wu.ac.jp; 4 Department of Sociology, University of Tokyo, 1-1-1, Yayoi, 5 Bunkyo-ku, Tokyo 113-8581, Japan; E-Mail: rahmenn@saito.co.jp

**Keywords:** dietary exposure, aflatoxin, ochratoxin A, rice, pasta, reduction

## Abstract

Mycotoxins are commonly present in cereal grains and are not completely destroyed during their cooking and processing. When mycotoxins contaminate staple foods, the risk for exposure becomes serious. In East Asia, including Japan, rice is consumed as a staple food, and with the increasingly Westernized lifestyle, the consumption of wheat has increased. The mycotoxins commonly associated with rice and wheat are total aflatoxin (AFL) and ochratoxin A (OTA), respectively. This study examined the retention of AFL and OTA during the cooking of rice and pasta. AFL was retained at 83%–89% the initial level after the cooking of steamed rice. In pasta noodles, more than 60% of the OTA was retained. These results show that AFL and OTA are relatively stable during the cooking process, suggesting that a major reduction in the exposure to these mycotoxins cannot be expected to occur by cooking rice and pasta. The estimated exposure assessment at the high consumer level (95th percentile) and the mycotoxin contamination level determined by taking into account these reductions in the present study should be useful for the establishment of practical regulations for mycotoxins in staple foods.

## 1. Introduction

Many mycotoxins are recognized to cause a variety of subacute and chronic health problems in humans. The production and occurrence of mycotoxins differ depending on the geographic and climatic conditions; however, these toxicants can never be removed completely from the food supply. Therefore many countries have defined levels of major mycotoxins in food, such as the maximum residue, that can be permitted for human consumption. The exposure assessment plays an important role in the establishment of this level. To accurately assess the exposure of foods, the impact of cooking and processing should therefore be taken into account.

Rice and wheat are staple food crops worldwide. Especially in many Asian countries, including Japan, rice is a staple food. Recently, due to the influence of Western culture, the consumption of wheat products, such as pasta, has been increasing in Japan, especially among younger people. 

Regarding mycotoxins, aflatoxin (AFL) contamination in rice and ochratoxin A (OTA) in pasta are thought to be the major problems [1,2,3,4]. AFL are a group of carcinogenic mycotoxins that cause liver cancer, with aflatoxin B1 (AFB1) exerting the strongest effect. The International Agency for Research on Cancer (IARC) classified AFL and AFB1 as class I agents [5]. OTA is a neurotoxic, immunosuppressive carcinogenic mycotoxin in humans and animals, and its target organ is the kidney [6]. The IARC has classified OTA as a group 2B agent. The Joint Export Committee of Food Additives (JECFA) has established provisional maximum tolerable weekly intake (PMTWI) at 100 ng/kg bw/day based on kidney toxicity [6]; however, the possibility of OTA-induced genotoxicity has recently been reported [7].

Our recent study of the surveillance results of AFL in Japan showed that the concentration of AFL in rice was under the limit of detection (LOD: 0.1 µg/kg) [8]. On the other hand, the surveillance results for OTA retained in food in Japan indicated that the occurrence of OTA in pasta was high (80%), and in young children, the exposure ratio for PMTWI of OTA was relatively higher than that for other age groups [9]. 

To estimate the dietary exposure to AFL and OTA from staple foods, such as rice and pasta, in Japan, in high consumers, we examined the reduction of AFL in rice and OTA in pasta after cooking. 

## 2. Results and Discussion

### 2.1. AFL in Rice after the Cooking Process

Since rice is eaten after steaming it in Japan, we employed the same procedure using the rice contaminated with aflatoxin naturally and the rice spiked with two different concentrations of AFL. The ratio of residual AFL for spiked test was calculated based on the following Equation:

The ratio of residual AFL (%) = (concentration of each or the total AFL in cooked rice spiked with AFL before steaming/concentration of each or the total AFL in cooked rice spiked with AFL after steaming × 100(1)

The recovery rates of the total amount of AFL were 90.2% for AFB1, 95.3% for AFB2, 74.1% for AFG1 and 81.5% for AFG2 when the rice was spiked with AFL before steaming.

**Figure 1:**
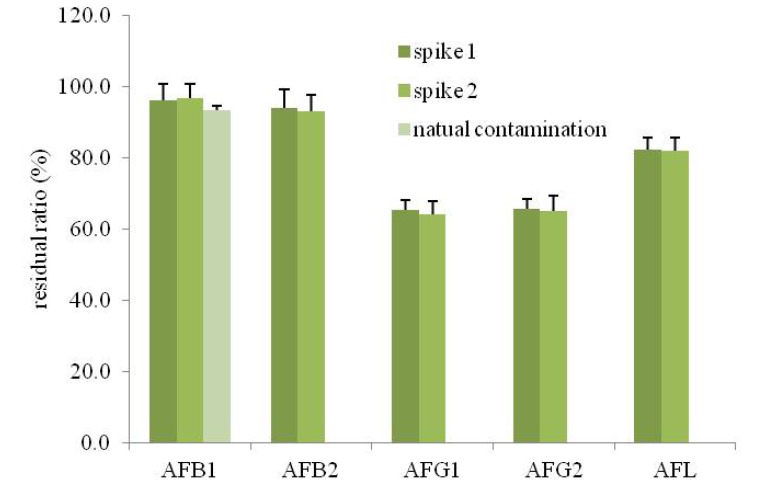
The residual ratio (%) of AFB1, AFB2, AFG1 and AFG2 in rice after cooking. The naturally contaminated rice and the artificially contaminated rice (spiked 1 and 2) were cooked as described in the Materials and Methods section. Spike 1 means that the polished rice was spiked with 2.5 ng/mL of AFB1, 1.875 ng/mL of AFG1 and 0.3125 ng/mL of AFB2 and AFG2. Spike 2 means that the polished rice was spiked with 5.0 ng/mL of AFB1, 3.75 ng/mL of AFG1 and 0.625 ng/mL of AFB2 and AFG2.

Figure 1 shows the ratio of the residual AFL in cooked rice. In the rice contaminated with AFB1 naturally, the residual ratio of AFB1 was 93.8% ± 1.2%. Since, the rice contaminated only AFB1, the effect of the cooking process on other AFs was examined using the rice contaminated with AFL artificially. In the first spiked experiment (2.5 ng/mL of AFB1, 1.875 ng/mL of AFG1 and 0.3125 ng/mL of AFB2 and AFG2), the residual ratios of AFB1, AFB2, AFG1, AFG2 and total AFL were 96.2% ± 4.6%, 94.0% ± 5.2%, 65.5% ± 2.5%, 65.7% ± 2.8% and 82.3% ± 3.4%, respectively. In the second spiked experiment (5.0 ng/mL of AFB1, 3.75 ng/mL of AFG1 and 0.625 ng/mL of AFB2 and AFG2), the same tendency was observed. These results indicated that AFB1 and AFB2 were stable during steaming, while AFG1 and AFG2 were degraded. Compared to the residue rate of AFB1 between naturally contaminated rice and artificially contaminated rice, there was no significant difference, suggesting that the spiked test would be used to estimate the residue rate of AFB2, AFG1 and AFG2.

Regarding the fate of AFL using naturally contaminated rice, Park *et al*. [10,11] reported that 31%–36% of AFB1 was lost during cooking at 160 °C for 20 min. In this study, we observed only a 7% reduction of AFB1. The reason for this difference was thought to be due to the differences in the cooking temperatures. Mohamadi *et al*. [12] examined the reduction of the total AFL (AFB1, AFB2, AFG1, AFG2) using a steaming-based rice cooker (Toshiba Corp., Tokyo, Japan) and reported that the average total AFL reduction was 24.8%. The reduction of the total AFL in the present study was almost the same as the result reported by Mohamadi *et al*. Hussain *et al*. [13] studied the reduction of AFB1 in polished basmati rice by boiling with water and microwave oven cooking using artificially contaminated rice. They reported that boiling and microwave oven cooking showed a reduction up to 84.0%–87.5% and 72.5% of initial contents, respectively. The extraction process of rice has been reported to be efficient [14]; however, this method is not popular in Japan. These results demonstrated that the total AFL content was retained at 82.3% of the original level under the ordinary cooking conditions used in Japan. 

### 2.2. Reduction of OTA in Pasta during the Cooking Process

Pasta is made from wheat and is consumed in many countries as a staple food. There have been many studies related to the reduction of Fusarium toxins, such as deoxynivalenol (DON), in pasta during cooking and processing [15,16,17], while there is limited information about the reduction of OTA. OTA has been reported to have possibility of genotoxicity [7,18], and the presence of OTA was detected in over 30% of all samples in Canada of cereals [19]. Our previous report showed the frequency of detection of OTA in dry pasta to be high in Japan [20]. In this study, to accurately estimate the exposure level from pasta, we prepared pasta using durum wheat spiked with OTA using the ordinary process of making pasta. The distribution and percent distribution of OTA in pasta and the broth in which it was boiled were calculated according to the method reported by Visconti *et al*. [15].

**Figure 2:**
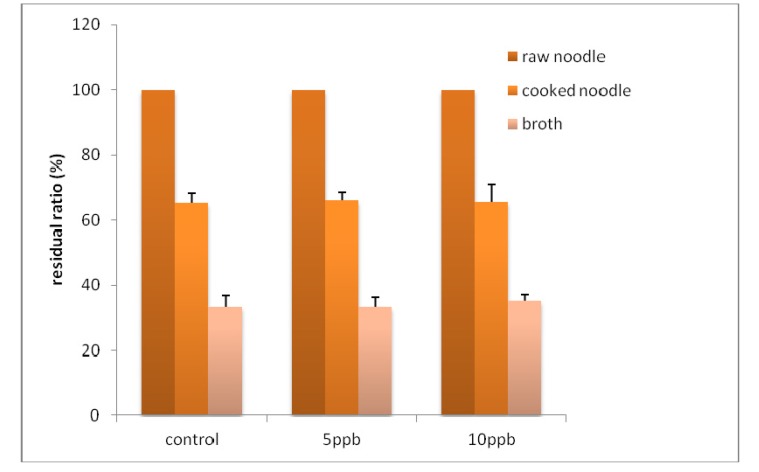
The residual ratio ofochratoxin A (OTA) in pasta after cooking. The pasta was made from durum wheat spiked with 0, 5 or 10 ng/g of OTA.

Figure 2 demonstrates the residual ratio of OTA in pasta and broth after cooking. The average retention in cooked pasta was 66.1% in the 5 ng/g and 65.8% in the 10 ng/g spiked samples, on a dry weight basis. The levels in the broth were 33.4% and 35.2% of the initial levels in the pasta spiked with 5 ng/g and 10 ng/g OTA, respectively. No significant difference in the OTA retention ratio between the 5 ng/g and 10 ng/g spiked pasta samples were observed. Therefore, the OTA was transferred to the broth during cooking, and the total amount of OTA in the pasta and the broth corresponded to the initial spiked amount. This suggests that OTA was not significantly changed or degraded during cooking. These results are in agreement with results obtained in the experiments about the reduction of DON [15]. Using Japanese-style noodles, which are made from wheat containing 8%–9% protein, the concentration of DON in the noodle decreased to 30% [21]. Compared to conventional pasta, the retention level was lower in the Japanese noodles. The reason why OTA was retained in the pasta may be related to the consistency and characteristics of durum semolina. This study suggested that OTA was hard to transfer to broth in pasta. 

### 2.3. Estimated Dietary Exposure and Risk Assessment of AFL and OTA in Rice and Pasta in Japan

Since people consume a larger amount of staple foods compared to other foods, the surveillance and dietary exposure assessment for mycotoxins contaminated in staple foods is important to prevent adverse health effects due to mycotoxins. 

There have been many studies about dietary exposure, which have focused on staple foods [22,23], but most of them did not consider the potential reduction of mycotoxin during the cooking process. The reduction that occurs during the milling and industrial processing is also important; however, in most cases, the surveillance and dietary exposure are performed on foods sold in retail shops. Therefore, an adequate understanding of the reduction during the cooking process is needed to more accurately estimate the dietary exposure. 

Since both rice and pasta are staple foods in Japan, the dietary exposure to AFB1 in rice and OTA in pasta in different age groups was calculated (Table 1). Since our surveillance study (2004–2005 fiscal years) demonstrated no samples with LOD in domestic rice, we could not estimate the degree of exposure to AFL from rice. As a result, in this study, we assumed the worst scenario regarding the exposure assessment of AFL. We used the consumption data for high consumers (95th percentile) and assumed that the contamination of AFB1 was also at the LOD.

The mean concentration of OTA in dry pasta in Japan was 0.48 µg/kg and 0.28 µg/kg in a four-year surveillance and a six-year surveillance study, respectively. In this study, we adopted the results of the six-year surveillance. As with AFB1 in rice, we used the consumption data for high consumers (95th percentile).

In the present situation, the dietary exposure to AFB1 in all foods contaminated (>LOQ) was 0.003 to 0.004 ng/kg bw/day, and the estimated cancer risk was 0.00005 at the 95th percentile in Japan [24]. Usually, it is thought to be of no practical use to estimate the risk related to food in which mycotoxin concentration is at LOD. However, since rice is a staple food that is frequently consumed in Japan, the LOD value is therefore considered to be important when carrying out a risk assessment for mycotoxin. Assuming that the AFB1 concentration of rice was at LOD, we therefore carried out a risk assessment for cancer.

From the experiments in this study, we demonstrated that the cooking processing factors for rice and pasta were 93.8% and 65.4%, respectively. These values were used in the exposure assessment as the cooking processing factor.

**Table 1:** Estimated exposure and risk assessment for AFB1 in rice and OTA in pasta in Japan.

Mycotoxin	Food	Age group	Consumption at the 95th percentile (g/kg bw/day)	Reduction during cooking (%)	Concentration of contamination ^a ^(µg/kg)	Dietary exposure to mycotoxin (ng/kg bw/day) at 95th percentile	PMTWI ^b ^(ng/kg bw/week)	Exposure/PMTWI (%)	Cancer risk ^d^	Margin of Exposure ^e^
Afatoxin B1	Rice	Toddlers and young children	24.96	93.8	0.1	2.34	- ^c^	-	0.040	107
		Older children	18.96	93.8	0.1	1.78	-	-	0.031	141
		Adolescents	14.16	93.8	0.1	1.33	-	-	0.023	188
		Adults	12.74	93.8	0.1	1.20	-	-	0.021	209
Ochratoxin A	Pasta	Toddlers and young children	1.17	65.4	0.28	0.21	100	1.50	-	91
		Older children	0.80	65.4	0.28	0.15	100	1.02	-	134
		Adolescents	0.37	65.4	0.28	0.07	100	0.48	-	286
		Adults	0.00	65.4	0.28	0.00	100	0.00	-	ND^ f^

^a^ The value was reported by surveillance results of AFL [8] and OTA [20]; ^b^ PMTWI:provisonal maximum tolerable weekly intake; ^c^ Since aflatoxin B1 is a genotoxin, the PMTWI could not be set; ^d^ Based on the calculated potency estimated (cancers/year per 100,000 people per ng/kg bw/day) by JECFA [25]; ^e^ Based on the calculated Benchmark dose lower limit 10% lower bound of AFB1 [26] (250 ng/kw bw/day) and OTA [27] (19.6 µg/kg bw/day); ^f^ ND:not determined.

Using this worst scenario, the risk assessment for AFB1 in Japan was performed for cancer risk [25]. We estimated that the dietary exposure to AFB1 in rice at the 95th percentile would be 1.20 to 2.34 ng/kg/day, so the cancer risk was estimated to be 0.021 to 0.040 cancer/100,000 people. The margin of exposure (MOE) was calculated to be 107–209 based on a benchmarked dose of the 10% lower limit (BMDL_10_) [26]. This result showed that, in Japan, the risk for AFB1 would be significant for high consumers, even though the overall AFL contamination was at LOD (0.1 ng/g). This suggests that the regulation of AFB1 for staple foods should be more restricted than for other foods.

The risk for OTA exposure from pasta evaluated as the ratio of PMTWI (exposure/PMTWI) and MOE based on the BMDL_10_ of developing renal cancer (19.6 µg/kg bw/day) was determined [27]. The ratio of the PMTWI was 0.48%–1.50%, and the risk in adults could not be calculated, because too little pasta was consumed. The MOE ranged from 91 to 286. These results demonstrated that the risk due to the consumption of pasta is low at present; however, among the most common staple foods, pasta is thought to be a major contributor of exposure to OTA in Japan. As a result, the monitoring of OTA contamination in dry pasta is thus considered to be important.

## 3. Experimental Section

### 3.1. Reagents

AFB1, AFB2, AFG1, AFG2 and OTA standards were purchased from Sigma-Aldrich Co. (St. Louis, MO, USA). Methanol and acetonitrile were high-performance liquid chromatography (HPLC) grade. Water was purified in a Milli-Q system (Millipore Co., Bedford, MA, USA). All other reagents were of the highest analytical grade available.

### 3.2. Preparation of Cooked Rice

The brown rice contaminated with aflatoxins naturally (TRILOGY, Analytical Laboratory, Washington, DC, USA) was used in this study. The aflatoxin concentration in the rice was aflatoxin B1 at 15.1 µg/kg. For the spiked tests, AFL solutions were prepared at a concentration of 100 ng/mL for AFB1, 75 ng/mL for AFG1 and 12.5 ng/mL for AFB2 and AFG2 in acetonitrile. A total of 20 g of washed-polished rice was spiked with 25 μL and 50 μL of the AFL solution and was kept for 1 h at room temperature. The spiked rice was then dipped in 37 mL of Milli-Q water and boiled at 100 °C for four minutes, then was subsequently steamed for 20 min in a commercial electric cooker (ZOUJIRUSHI Corporation, NP-GD05, Osaka, Japan). After cooking, the rice was added to a four-fold volume of Milli-Q water and homogenized (3000 rpm, 5 min). The homogenized sample was extracted with a 16-fold volume of methanol. The extract was filtered and diluted with a 6.25-fold volume of PBS and then was applied to an immune-affinity column (AFLAKING, HORIBA, Ltd., Kyoto, Japan), according to a method described in previous report [8]. The eluent was dried with N_2_, dissolved with acetonitrile-water (1:9) and then was analyzed.

### 3.3. Preparation of Cooked Pasta

The durum semolina used in this study (Leone G; Nisshin Flour Milling Inc., Tokyo, Japan) was naturally contaminated with 2.2 ng/g of OTA. The wheat and water were premixed at 30% absorption at a lowest speed in a vertical mixer using a hook-type mixing paddle for 5 min. OTA that had previously been dissolved in ethanol (final concentrations were 5 ng/g and 10 ng/g) was added to the water used for mixing the wheat. The control wheat was prepared using dried pasta spiked with ethanol only. Dried pasta was processed in a MAC 60 laboratory-scale extrusion press (ITALPAST S.r.I. Parma, Italy) and dried in a PR-4KP environmental chamber (ESPEC Corp., Osaka, Japan). The premixed dough was transferred to a final mixing chamber of the MAC 60 extrusion press and extruded under vacuum (82,000 Pa). A 42 hole, 0.8 × 6.0 mm fettuccine die was used for extrusion. The fresh pasta was dried in the PR-4KP environmental chamber at 80 °C for 7 h. Both the control and spiked pasta samples (10 g) were boiled for 6 min in 400 mL of boiling water with 0.5 g NaCl. A total of 40 mL of extract solution (acetonitrile and water (6:4)) was added to all pasta samples, which were then homogenized at 3000 rpm for 3 min. The extract was centrifuged at 3000 rpm for 5 min, and the supernatant was brought to a final volume of 100 mL with PBS. After filtration, 10 mL of the samples were applied to an immune-affinity column (OCHRAKING, HORIBA, Ltd., Kyoto, Japan), according to the previous report [9].

The eluent was dried up with N_2_, dissolved with acidic acid-water and then analyzed. The broth removed after the pasta was completely cooked and was concentrated until it became a slurry in order to analyze. The slurry was then extracted with a four-fold volume of the extract solution and cleaned up with the immune-affinity column. All cooking experiments were performed in triplicate. All samples were analyzed in triplicate.

### 3.4. Determination of AFL and OTA

AFL was quantified by an HPLC method with a fluorescence detector (FD, excitation, 360 nm; emission, 450 nm), as described previously [8]. The analytical column (4.6 mm by 250 mm by 5 μm; Inertsil ODS-3V, GL Sciences, Inc., Tokyo, Japan) was kept at 45 °C, with a mobile phase of acetonitrile-methanol-water (1:3:6) at a flow rate of 1.0 mL/min for trifluoroacetic acid (TFA) derivatization or with acetonitrile-methanol-water (2:3:5) at a flow rate of 0.7 mL/min for the photochemical reactor. The LODs for AFB1, AFB2, AFG1 and AFG2 in cooked rice by HPLC-FD were 0.1 ng/g.

OTA was also analyzed by HPLC-FD (excitation, 333 nm; emission, 460 nm), as described previously [10]. The analytical column (4.6 mm by 250 mm by 5 mm; Inertsil ODS-3V, GL Sciences, Inc., Tokyo, Japan) was kept at 45 °C with a mobile phase of acetonitrile-water-acetic acid (55:43:2) at a flow rate of 1.0 mL/min. The detection limit for OTA in pasta by HPLC-FD was 0.1 ng/g, and the recovery rate was 97.5%. LODs and limits of quantification were calculated based on signal-to-noise ratios of 3:1 and 10:1, respectively.

### 3.5. Simulation of the Estimated Exposure and Risk Assessment of Rice and Pasta in Japan

The food consumption data originated from the National Health and Nutrition Survey and was conducted from 2007 to 2010. The survey followed consumption over a period of two consecutive days from 17,827 individuals in four different age groups: toddlers and young children (1–6 years old), older children (7–14 years old), adolescents (15–19 years old) and adults (>20 years old). The distribution of the consumption of 105 food items in the four different age groups was simulated by fitting the log-normal distribution to the consumption data for steamed rice and pasta and then multiplying the distribution of the consumption of these foods based on the data in the database for each food product. 

The parameters used in this table were calculated by the following Equation:

The dietary exposure to mycotoxin = consumption at the 95th percentile × contamination concentration × cooking processing factor(2)

## 4. Conclusions

In this study, to accurately estimate the dietary exposure due to staple foods in Japan, the cooking factor for steamed rice and pasta were calculated and used for new risk estimate formulas. The fate of AFL in steamed rice and OTA in pasta during cooking were investigated using rice and pasta spiked with various concentrations of AFL or OTA. The results indicated that steaming rice led to an approximately 18% loss of AFL; however, the AFB1 concentration was not significantly reduced. While the OTA in pasta could be released into water, about 60% of it was retained in the boiled noodles. Taking these residue ratios into account as cooking factors and by using the surveillance results, we estimated the exposure and risk assessment for AFL and OTA in Japan, assuming that the concentrations of AFL were at LOD and that the concentration of OTA was the mean concentration detected in pasta during a recent surveillance study [8].

Based on the results of the present study, even though the concentration of AFL was at LOD, if the toxicant was contained in a staple food, such as rice, there was a notable increase in the cancer risk due to the high consumption of that food. We recommend that for staple foods, the maximum residue level should be reconsidered and that the permissible level for staple foods should be more restricted than for other foods.
